# AtELP4 a subunit of the Elongator complex in *Arabidopsis*, mediates cell proliferation and dorsoventral polarity during leaf morphogenesis

**DOI:** 10.3389/fpls.2022.1033358

**Published:** 2022-10-21

**Authors:** Sang Eun Jun, Kiu-Hyung Cho, Muhammad Aamir Manzoor, Tae Young Hwang, Youn Soo Kim, Raffael Schaffrath, Gyung-Tae Kim

**Affiliations:** ^1^ Department of Molecular Genetics, Dong-A University, Busan, South Korea; ^2^ School of Life Sciences, Anhui Agricultural University, Hefei, China; ^3^ Graduate School of Applied Bioscience, Dong-A University, Busan, South Korea; ^4^ Institut für Biologie, Fachgebiet Mikrobiologie, Universität Kassel, Kassel, Germany

**Keywords:** AtELP4, cell proliferation, DRL1, Elongator complex, leaf polarity

## Abstract

The Elongator complex in eukaryotes has conserved tRNA modification functions and contributes to various physiological processes such as transcriptional control, DNA replication and repair, and chromatin accessibility. *ARABIDOPSIS* ELONGATOR PROTEIN 4 (AtELP4) is one of the six subunits (AtELP1–AtELP6) in *Arabidopsis* Elongator. In addition, there is an Elongator-associated protein, DEFORMED ROOTS AND LEAVES 1 (DRL1), whose homolog in yeast (Kti12) binds tRNAs. In this study, we explored the functions of AtELP4 in plant-specific aspects such as leaf morphogenesis and evolutionarily conserved ones between yeast and *Arabidopsis*. ELP4 comparison between yeast and *Arabidopsis* revealed that plant ELP4 possesses not only a highly conserved P-loop ATPase domain but also unknown plant-specific motifs. ELP4 function is partially conserved between *Arabidopsis* and yeast in the growth sensitivity toward caffeine and elevated cultivation temperature. Either single *Atelp4* or *drl1-102* mutants and double *Atelp4 drl1-102* mutants exhibited a reduction in cell proliferation and changed the adaxial–abaxial polarity of leaves. In addition, the single *Atelp4* and double *Atelp4 drl1-102* mutants showed remarkable downward curling at the whole part of leaf blades in contrast to wild-type leaf blades. Furthermore, our genetic study revealed that *AtELP4* might epistatically act on *DRL1* in the regulation of cell proliferation and dorsoventral polarity in leaves. Taken together, we suggest that AtELP4 as part of the plant Elongator complex may act upstream of a regulatory pathway for adaxial–abaxial polarity and cell proliferation during leaf development.

## Introduction

Leaf formation in plants requires intricate and multiple mechanisms to determine various factors including leaf size and shape, phyllotaxis, heteroblasty, and structural asymmetry. These mechanisms are managed through genetic programs adapted by leaf function and environmental cues during evolutionary progression. To accomplish leaf development, plants undergo dynamic sequences of regulatory processes: from loss of meristematic stem cell identity, leaf initiation, establishment of leaf polarity (adaxial–abaxial, medial–lateral, and distal–proximal axis), cytoplasmic growth, cell proliferation and expansion, endoreduplication, cell type-specific differentiation such as guard cells, vascular tissue, and trichomes, to mature leaves (Kalve et al., 2014). During adaxial/abaxialization among these processes, leaves construct the adaxial side, which is adjacent to the shoot apical meristem (SAM), the upper sun-exposed side in mature leaves, and the abaxial side, which is farther from SAM, the lower shaded side in mature leaves. Leaf adaxial/abaxialization specializes leaf external and internal structure according to their function; the adaxial portion is built up into compactly packed and chloroplast-rich cells and more trichomes for optimal light capture and defense, whereas the abaxial portion is built up into loosely packed cells and more guard cells and stoma for gas exchange. The vascular bundle has also discrimination between adaxial and abaxial sides, showing different positioning of xylem and phloem cells. Therefore, the acquisition of adaxial/abaxial identity in leaf structure is necessary for the performance of leaf function. Numerous studies have shown that the expression of *class III homeodomain-leucine zipper* (*HD-ZIP III*) genes, *PAHBULOSA* (*PHB*), *PHAVOLUTA* (*PHV*), and *REVOLUTA* (*REV*) determines the identity of the adaxial domain (McConnell et al., 2001), while the expression of *YABBY* (*YAB*) and *KANADI* (*KAN*) gene families determines the identity of the abaxial domain (Eshed et al., 2004). In addition, the expression of adaxial- or abaxial-determinant genes is regulated not only by each other through negative feedback (Tsukaya, 2013) but also by small RNAs and auxin (Pekker et al., 2005; Alvarez et al., 2006).

The growth imbalance between the adaxial side and the abaxial side causes defects in leaf flatness, and leaf epinasty or hyponasty (upward or downward curling, respectively) (Sandalio et al., 2016). These morphological changes are triggered by alteration in auxin accumulation or gradient (Enders and Strader, 2015). However, it is not well known how auxin controls the leaf flatness or the growth difference between the adaxial/abaxial sides.

Apart from the differential transcription of protein-encoding genes, an emerging mechanism to regulate protein levels is translational modulation. The latter involves the modification of tunable mRNA transcripts (MoTT) with their decoding coupled to tRNA modifications, in particular anticodon wobble uridine (U_34_) bases. The Elongator complex was originally identified as a transcription factor in yeast (*S. cerevisiae*) (Otero et al., 1999; Wittschieben et al., 1999). Meanwhile, however, it is widely accepted that the primary and conserved role of Elongator from yeast to plants and humans lies with tRNA modification and mRNA translation (Otero et al., 1999; Hawkes et al., 2002; Huang et al., 2005; Mehlgarten et al., 2010; Nelissen et al., 2010; Johansson et al., 2018; Nakai et al., 2019; Abbassi et al., 2020). Accordingly, the Elongator complex (Elp1–Elp6) is key to a pathway that adds 5-carboxy-methyl (cm^5^) groups to several tRNA anticodons (Selvadurai et al., 2014; Glatt et al., 2016; Lin et al., 2019; Dauden et al., 2019). Among others, these can be further modified to 5-methoxy-carbonyl-methyl (mcm^5^) groups or 2-thio derivatives (mcm^5^s^2^) thereof in concert with U_34_ methylase and thiolase activities (Schaffrath and Leidel, 2017; Johansson et al., 2018; Sokołowski et al., 2018).

The Elongator complex consists of two subcomplexes, each comprising three subunits: Elp123 and Elp456 (Dauden et al., 2019). In yeast, there is an additional protein (Kti12/Tot4), which associates with Elongator (Frohloff et al., 2001; Mehlgarten et al., 2017). It binds tRNA and is essential for the tRNA modification activity, which resides in the Elongator catalytic subunit Elp3 (Svejstrup, 2007; Glatt et al., 2016; Krutyhołowa et al., 2019; Abbassi et al., 2020). Elongator components in eukaryotes are structurally conserved (Dauden et al., 2017a; Dauden et al., 2017b), and previous studies had reported that in yeast, two copies each of Elp4, Elp5, and Elp6 form a RecA-ATPase-like ring structure with roles in substrate recognition during Elongator-mediated tRNA modification (Glatt et al., 2012; Glatt et al., 2016; Dauden et al., 2017a; Dauden et al., 2017b). Despite their similarity in structure and tRNA modification activity, Elongator complexes from yeast, mammals, and plants may functionally differ in various physiological processes and contexts (Nelissen et al., 2005; Close et al., 2006; Chen et al., 2009; Nelissen et al., 2010; Nakai et al., 2019).

Previously, we isolated and characterized *Arabidopsis DEFORMED ROOTS AND LEAVES 1* (*DRL1*), a gene homologous to yeast *Kluyveromyces lactis TOXIN INSENSITIVE 12/TOXIN TARGET 4* (*KTI12/TOT4*), which encodes an Elongator-associated tRNA binding protein (Mehlgarten et al., 2017; Krutyhołowa et al., 2019). DRL1 shows a partially conserved function to yeast Kti12 and is involved in the regulation of leaf polarity and cell proliferation of leaves of *Arabidopsis thaliana*. In this study, we characterized AtELP4 functions. Although we could not detect binding between AtELP4 and DRL1 or other individual plant Elongator subunits and DRL1, we found that an *Atelp4* mutant showed narrow and abaxialized leaves very similar to those of a *drl1-102* mutant. We suggest that AtELP4 as part of the Elongator complex in *Arabidopsis* plays critical roles in the establishment of leaf polarity, especially adaxialization through a collaboration with DRL1 and regulation of leaf cell proliferation.

## Materials and methods

### Phylogeny relationship, domain, and motif analysis

All ELP4 full-length protein sequences from various eukaryotes identified and verified conserved domain through Pfam (https://pfam.xfam.org/) (Finn et al., 2006), NCBI Conserved Domain Database (CDD) database (https://www.ncbi.nlm.nih.gov/Structure/cdd/cdd.shtml), and SMART tool (http://smart.emblheidelberg.de) (Letunic et al., 2012). In addition, identified *ELP4* genes were aligned using ClustalX software with default parameters (Thompson et al., 1997). Finally, the phylogeny was constructed by using the neighbor-joining method (NJM). The amino acid sequences of ELP4 proteins used in the alignment were retrieved from the NCBI GenBank database (https://www.ncbi.nlm.nih.gov/). The GenBank accession numbers of the amino acid sequences are *A. thaliana* (Q9C778.1), *Ostreococcus tauri* (XP_003082942.1), *Marchantia polymorpha* subsp. *ruderalis* (BBN04784.1), *Physcomitrella patens* (XP_024385693.1), *Selaginella moellendorffii* (XP_024534079.1), *Amborella trichopoda* (XP_020520905.1), *Nymphaea colorata* (XP_031482638.1), *Zea mays* (NP_001131483.1), *Sorghum bicolor* (XP_021304923.1), *O. sativa* Japonica Group (XP_015643899.1), *Brachypodium distachyon* (XP_003563291.1), *Phalaenopsis equestris* (XP_020577487.1), *Macadamia integrifolia* (XP_042486942.1), *Rosa chinensis* (XP_040364853.1), *Glycine max* (KAH1257688.1), *Medicago truncatula* (XP_013443887.1), *Gossypium hirsutum* (XP_016734795.1), *Hibiscus syriacus* (KAE8729835.1), *Vitis vinifera* (RVW57326.1), *Helianthus annuus* (XP_035832566.1), *Manihot esculenta* (XP_021624621.1), *Cucumis sativus* (XP_031743242.1), *Ipomoea nil* (XP_019185341.1), *Nicotiana tabacum* (XP_016448924.1), *Brassica napus* (XP_022558390.1), *Salvia hispanica* (XP_047969651.1), *Solanum lycopersicum* (XP_025883858.1), *S. cerevisiae* (NP_015224), *Schizosaccharomyces pombe* (Q9USP1), *K. lactis* (XP_455048), *Dictyostelium discoideum* (Q54XS0.1), *Hydra vulgaris* (XP_047135159.1), *Caenorhabditis elegans* (NP_001370473.1), *Strongylocentrotus purpuratus* (XP_030844920.1), *Aplysia californica* (XP_005105912.1), *Drosophila melanogaster* (Q9VMQ7), *Xenopus laevis* (NP_001088363.1), *Danio rerio* (NP_001017638.1), *Mus musculus* (NP_076365.2), and *Homo sapiens* (KAI2559206.1). Subsequently, conserved motifs of ELP4 in plants, yeasts, and animals were identified by using the MEME online tool (http://meme-suite.org/tools/meme) with the other default parameters and five maximum numbers of motifs (Bailey et al., 2015).

### Syntenic relationship analysis

Sequence similarity of *ELP4* genes between the plant and yeast was carried through circoletto software (http://tools.bat.infspire.org/circoletto/) (Darzentas, 2010). *ELP4* gene sequences were used as queries against plants and yeast. The E-value was maintained constant at 1 × 10^−10^ and displays the sequences that produced hits based on percentage (%) identity (blue color 70%, green color 80%, orange color 90%, and red color 100%) with other default parameters.

### *Cis*-acting elements and gene ontology annotation analysis

The promoter sequences (ATG start codon with 1,500 bp) of *ELP4* genes were estimated through the online PlantCARE database (https://bioinformatics.psb.ugent.be/webtools/plantcare/html/) (Lescot et al., 2002). CELLO2GO was used to identify the gene ontology (GO) annotations of the whole *ELP4* genes. The CELLO platform (http://cello.life.nctu.edu.tw/) uses hierarchical vocabularies to connect genes and GO terms. Functional enrichment analysis of *ELP4* genes was carried out by using DAVID 6.8 (http://david.ncifcrf.gov/) online tools. The gene ontology enrichment was categorized into three main groups: biological process (BP), cellular component (CC), and molecular function (MF).

### Yeast strains, media, and general methods

Yeast strains were from *S. cerevisiae* wild-type BY4741 (*MAT*a *his3*Δ1 *leu2*Δ0 *met15*Δ0 *ura3*Δ0) and *ELP4/KTI9/TOT7* deletion mutant Y02150 (As BY4741, but *elp4Δ*) (Brachmann et al., 1998). At 30°C, yeast strains were consistently cultivated on standard rich (YPD) and minimal (SC minimal) growth media. For the test of thermosensitivity and caffeine sensitivity, yeast stains grew on YPD media with or without caffeine at 30°C or 38°C.

### Yeast complementation test

*Arabidopsis* and yeast ELP4 coding regions were amplified using the *Eco*RI site at the 5′ end and *Bam*HI site at the 3′ end to validate *AtELP4* in yeast *elp4*Δ deletion mutant by using the following primers *Eco*RI-*AtELP4*‐for (5′‐GGA ATT CCA TGG CTG CAC CAA ACG TTC GTA G‐3′) and *Bam*HI-*AtELP4*-rev (5′‐CGG GGA TCC CGT CAA AAA TCT AGT GCT CCG G‐3′), and DNA fragment was cloned into pBluescript SK(-) vector. The *AtELP4* fragments eluted from *Eco*RI and *Bam*HI digestion were subcloned into the *Eco*RI–*Bgl*II site of the pTU1 vector, which were overexpressed under the control of the *TDH3* promoter and *CMK1* terminator in the pTU1 vector. Yeast *elp4Δ* cells were transformed with pTU1 empty vector and *AtELP4*-harboring pTU1 using electroporation. Transformants were selected on a minimal medium lacking uracil (SD-U) (Jun et al., 2015).

### Plant materials and growth conditions

Individual *Arabidopsis* homozygous T-DNA insertion mutants (Columbia-0 (Col-0) background) in *AtELP4* (At3g11220, SALK 079193/*Atelp4*) and *DRL1* (At1g13870, SALK 056915/*drl1-102*) were obtained from the *Arabidopsis* Biological Resource Center (ABRC; https://abrc.osu.edu). The homozygote genotype of insertion mutants was confirmed by amplification of T-DNA in T-DNA inserted genomic DNA region using T-DNA and gene-specific primers. The double mutant was germinated by crossing single mutants, isolated in the F2 progeny, and confirmed by the abovementioned PCR-based method.

Plants were grown in a Murashige–Skoog (MS) media containing 0.43% MS medium salt mixture, 2% sucrose, and 0.2% gellan gum (pH 6.3) and transferred to a mixture of soil, vermiculite, and pearlite (2:1:1) under long-day conditions (16-h light/8-h dark, 50–100 μE/m^2^/s) at 22°C. Seeds were surface-sterilized, vernalized for 3 days at 4°C, and then germinated on MS media (Jun et al., 2015).

### Anatomical analyses

The mature third rosette leaves were collected from plants on 21 DAS and fixed with fixation solution (5% (v/v) acetic acid, 5% (v/v) formaldehyde, and 45% (v/v) ethanol in water) under a vacuum. Then, they were dehydrated and cleared as described (Jun et al., 2019). For transverse sectional analysis of leaves, the widest region of dehydrated leaves was embedded in Technovit 7100 resin (Kulzer & Co. GmbH, Wehrheim, Germany) and sliced transversely by using a microtome (Jun et al., 2019). Slices were stained with toluidine blue [1% toluidine blue in phosphate-buffered saline (PBS)], dried, and then observed.

Leaf length and width were measured using from tip to base and the widest part of the leaf blade from 10 mature third rosette leaves of individual plants on 21 DAS. Palisade cells in cleared leaves and transverse sectional slices were observed by light microscopy (Axioskop2, Carl Zeiss, Germany). Cell numbers were counted in the region from mid-vein to leaf margin of the widest region in leaves, and cell size and intercellular airspace were measured from palisade cells of the central region between mid-vein and leaf margin at the widest region in leaves from six mature third rosette leaves of the individual plant on 21 DAS.

The NIH IMAGE software ImageJ was used to analyze the data, and the Statistical Package for the Social Sciences program (SPSS 13.0, SPSS Inc. Chicago) was used for statistical analysis.

### Measurement of chlorophyll content and photosynthetic efficiency

To estimate total chlorophyll, fresh weight 0.1 g of leaf blade was taken from 28-day-old plants and soaked in 2 ml of 90% aqueous acetone at 25°C in the dark for 12 h until being cleared by centrifugation for 1 min at 5,000 rpm. The absorbance of the supernatant was measured at wavelengths 645, 663, and 750 nm using a spectrophotometer. Chlorophyll *a* and *b* contents were estimated according to the formula of Ritchie (Ritchie, 2006).

Photosynthesis efficiency was calculated from 10 plants by measuring chlorophyll fluorescence imaging with the Handy FluorCam fluorescence imaging equipment (Photon Systems Instruments, Brno, Czech Republic). Subsequently, *Fv*/*Fm* value was estimated from individual plants using FluorCam7 software according to the manufacturer’s instructions (Arena et al., 2020).

### RNA isolation and qRT-PCR analysis of gene expression

Total RNA was isolated from rosette leaf blades of 21-day-old plants and yeast using the RNeasy mini kit (Qiagen, Hilden, Germany), and cDNA was synthesized by reverse transcription using the Reverse TraAce-a-First strand cDNA synthesis kit (TOYOBO, Tokyo, Japan). The quantitative RT-PCR (qRT-PCR) was performed using SYBR^®^ Premix Ex TaqTM II (TAKARA, Otsu, Japan) and Bio-Rad CFX96TM Real-Time System (Bio-Rad, Hercules, CA, USA) with three technical replicates. Primers were designed by Primer-BLAST (https://www.ncbi.nlm.nih.gov/tools/primer-blast/index.cgi) for qRT-PCR and are listed in **Supplementary Table 5**. PCR thermocycles were run using the following conditions: denaturation at 95°C for 5 min, followed by 40 cycles of denaturation at 95°C for 30 s, annealing at 55°C for 30 s, and extension at 72°C for 30 s. The normalized expression level of each gene was calculated using the *ΔΔCq* method and *β-tubulin* (*TUB4*) gene for *Arabidopsis*, and *S. cerevisiae ACTIN 1* (*ScACT1*) gene for yeast was used as a control.

### DNA ploidy analysis

The mature third rosette leaf of plants on 21 DAS was harvested, and nuclei of leaves were extracted in Otto buffer (Otto, 1992; Doležel and Göhde, 1995) by chopping. Isolated nuclei were stained with propidium iodide (PI). Finally, DNA ploidy was analyzed with the flow cytometer, Cytomics FC500 flow cytometer (Beckman Coulter, Brea, CA, USA).

## Results

### Phylogeny relationship, evolutionary, conserved motif, and shared plant-specific domain analysis

As mentioned above, the Elongator complex was originally identified as a transcription factor in yeast (*S. cerevisiae*) (Otero et al., 1999; Wittschieben et al., 1999). Elongator components in eukaryotes are structurally conserved (Dauden et al., 2017a,b; Otero et al., 1999). Petrakis et al. (2005) suggested a weak, salt-labile interaction between yeast Kti12 and Elp4, although they did not prove the presence of Elp4 in the highly purified Kti12. Based on these findings, we attempted to examine the functions of *AtELP4* and its genetic interaction with *DRL1*, a gene homologous to yeast *KTI12*. At first initially, we performed an amino acid alignment and phylogenetic analysis of ELP4 from various eukaryotes including yeast, human, and plants using MEGA7 software. ClustalX pairwise alignment of AtELP4 amino acid sequences with ELP4s from several species indicated that AtELP4 proteins displayed approximately 60% identity with plant ELP4 proteins, but only about 20% identity was observed with yeast or animal Elp4 proteins. In detail, AtELP4 was approximately 65% identical with other dicot plant ELP4 proteins (*V. vinifera*, *C. sativus*, *G. max*, and *S. lycopersicum*) and about 57% identical with monocot plant ELP4 proteins (*O. sativa* and *Z. mays*). Moreover, AtELP4 was just 20% identical to yeast or human Elp4 proteins (**Supplementary Table 1**).

However, we also performed the structural analysis of *ELP4* using NCBI (Conserved Domain Database) (https://www.ncbi.nlm.nih.gov/cdd) to find conserved motifs of the ELP4 from various eukaryotes. ELP4 from eukaryotes shared PAXNEB domain, which is found in the histone acetyltransferase complex of RNA polymerase II holoenzyme. Since human and mouse *PAXNEB* genes were identified as yeast *ELP4* homolog due to their significant homology (Winkler et al., 2001); PAXNEB domain (accession no. PF05625, https://www.ebi.ac.uk/interpro/entry/pfam/PF05625/) became a common/representative domain of an RNA polymerase II Elongator protein subunit. PAXNEB domain is known as the P-loop ATPase motif of RecA or RAD families, which catalyze recombination reactions using ATP-dependent DNA binding and DNA-dependent ATP hydrolase activities (Ponting, 2002; Nelissen et al., 2005; Glatt et al., 2012). Especially, PAXNEB domain in plant ELP4 homologs was conserved with KaiC-like domain (accession no. CD01124, https://www.ncbi.nlm.nih.gov/Structure/cdd/cd01124), which was found in KaiC, a circadian clock protein in cyanobacteria and possesses autophosphorylation activity (E-value < 0.1).

**Figure 1:**
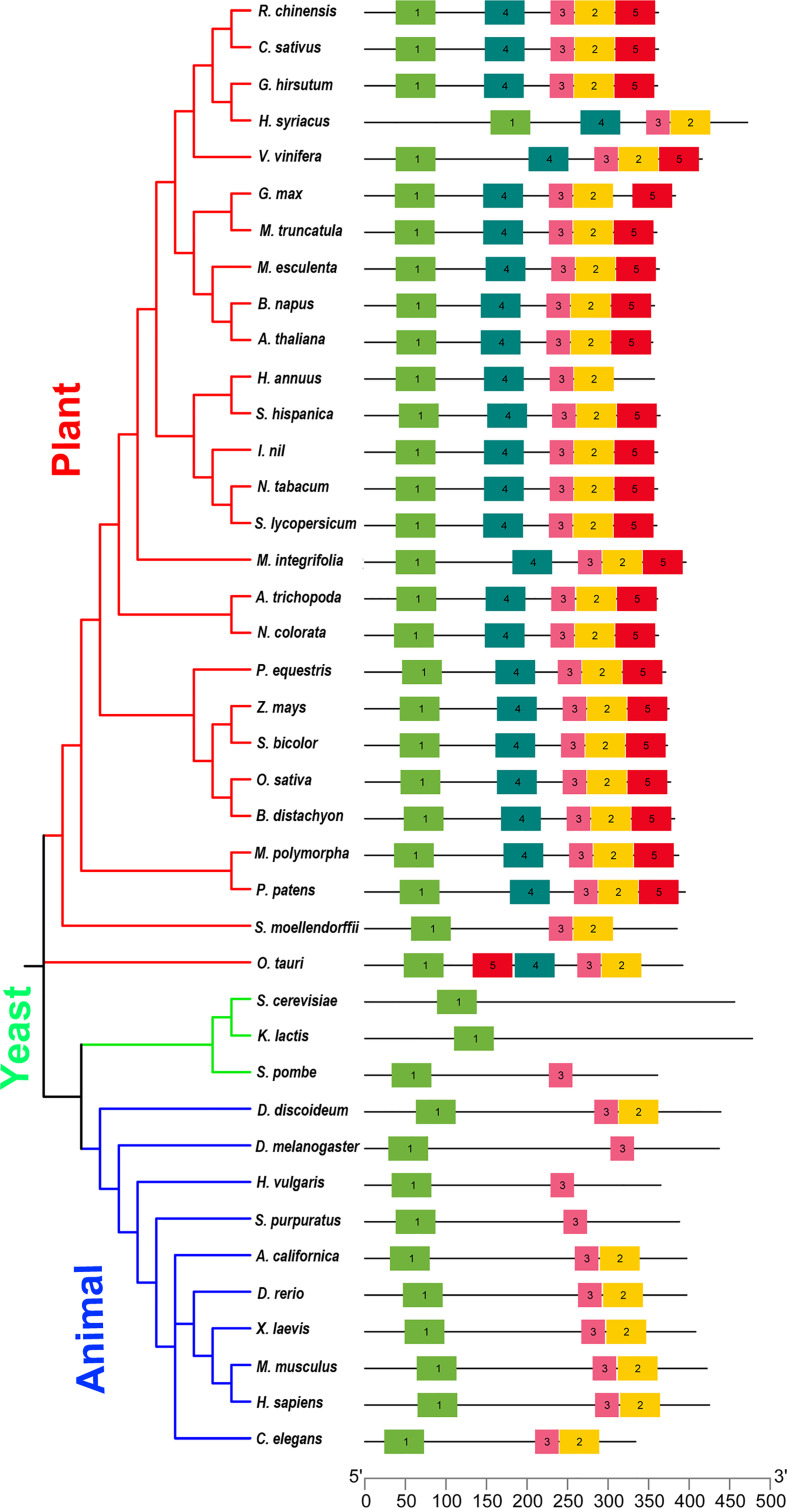
The phylogeny and conserved motif analysis and classified into three groups: plant, yeast, and animal. ELP4 protein motif composition is represented by distinct colored boxes with appropriate motif numbers and figure legends at the top.

MEME online tool was used to uncover unknown motifs among ELP4 from plants, yeast, and animals (http://meme-suite.org/). A minimum of five motifs were used in the motif analysis along with other default parameters. A phylogenetic tree was built in order to better comprehend the makeup of the 27 plants, 3 yeast, and 10 animal species. The phylogeny was classified into three groups (plants, animals, and yeast) (**Figure 1**). We investigated the conserved motif distribution on ELP4. ELP4 proteins in the three groups share the same conserved motifs, which supports the phylogenetic tree conclusions (**Figure 1**). However, ELP4 proteins contain a variety of conserved motifs in each group. Motif 1 was identified as conserved motifs and could be traced in all groups (plant, animal, and yeast). Motifs 2 and 3 were found in plants and animals, not in yeast, showing specific features in multicellular organisms. Interestingly, motif 4 and motif 5 were present only in ELP4 of plants, indicating that these families have some unique evolutionary processes and functionalities (**Figure 1** and **Supplementary Table 2**). These results of phylogenic analysis and conserved motif search revealed that *ELP4* might play not only representative roles but also species-specific roles according to organisms, although *ELP4* homologs are evolutionarily conserved.

### Syntenic relationship between plant and yeast

**Figure 2:**
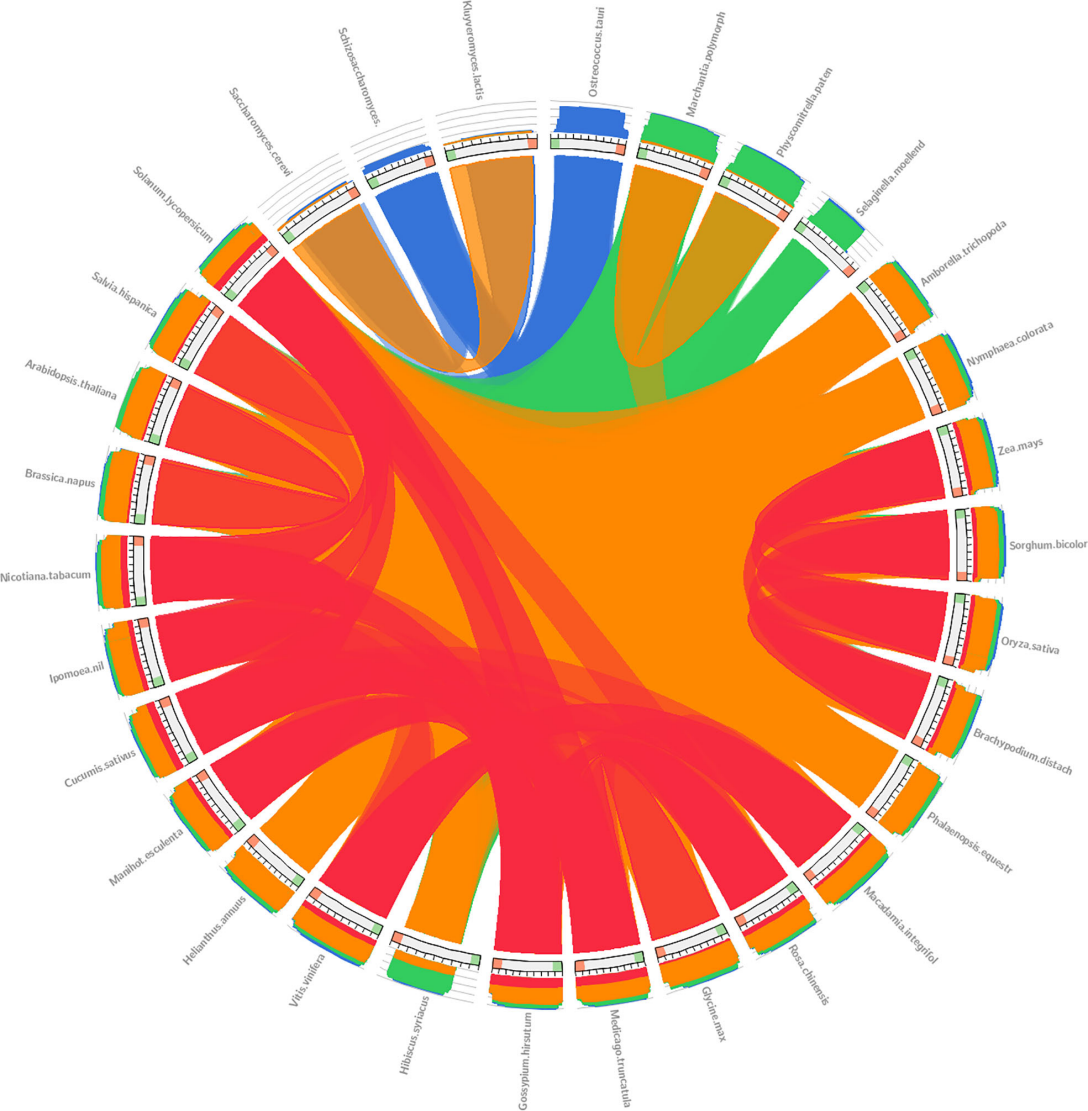
The circos tool was used to determine the synteny relationship of *ELP4* genes between the plant and yeast. The colors blue, green, orange, and red represent ≤50%, ≤70%, ≤90%, and >90% similarity, respectively.

We evaluate the collinearity relationship among plant and yeast species. The 27 plants (*A. trichopoda*, *A. thaliana*, *B. distachyon*, *B. napus*, *C. sativus*, *G. max*, *G. hirsutum*, *H. annuus*, *H. syriacus*, *M. integrifolia*, *M. esculenta*, *M. polymorpha* subsp *ruderalis*, *M. truncatula*, *N. tabacum*, *N. colorata*, *O. sativa*, *P. equestris*, *P. patens*, *R. chinensis*, *S. hispanica*, *S. moellendorffii*, *S. lycopersicum*, *S. bicolor*, *V. vinifera*, *Z. mays*, *O. tauri*, and *I. nil*) and three yeast (*S. pombe*, *S. cerevisiae*, and *K. lactis*) were subjected to microsynteny analysis to better understand the evolutionary and origin mechanism of *ELP4* genes. Synteny analysis in plants and yeast revealed a substantial association between duplication, expression, gene evolution, triplication, and function. *A. thaliana* gene sequence demonstrated synteny with the *B. napus* gene sequence, with the red color indicating that these genes have 100% similarities (**Figure 2**). Furthermore, yeast (*S. pombe*, *S. cerevisiae*, and *K. lactis*) had synteny with each other and among plants, which was identical in orange (90%) and blue (70%) colors (**Figure 2**). *ELP4* genes belonging to plants are mostly connected *via* red lines, showing more than 100% identity among plants. As yeast is considered the progenitor of the plants, the same thing was supported by our results where some plant sequences were 70% identical to yeast genes and are shown in blue color in **Figure 2**. Our result suggests that there are collinearity relationships between the different plant and yeast gene sequences, suggesting a potential evolutionary mechanism.

### *Cis*-acting elements analysis

**Figure 3:**
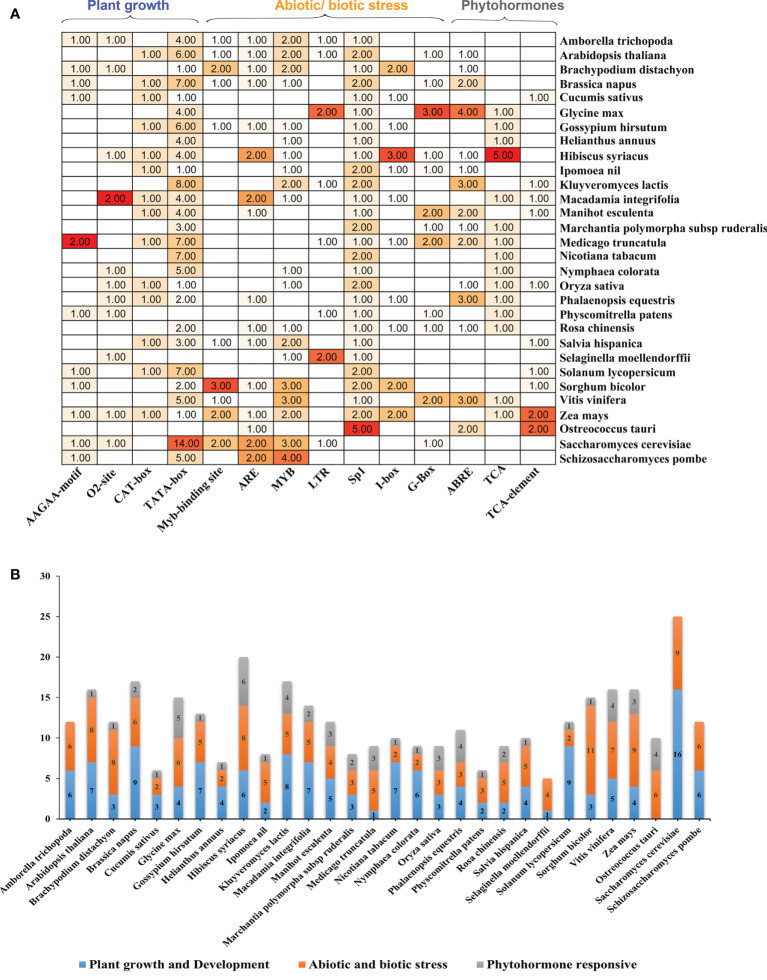
*Cis*-acting elements in *ELP4* promoter of various plants and yeast. **(A)** *Cis*-acting elements with functional similarity on the upstream region of *ELP4* genes are demonstrated by multiple colors. **(B)** In each category (plant growth development, abiotic/biotic stress, and phytohormone responsive), present *cis*-acting elements represent with different colors.

The potential *cis*-acting factors were identified by using PlantCARE to better comprehend the expression control mechanism of *ELP4* genes in plants and yeasts (**Supplementary Table 3**). These *cis*-acting elements were categorized mainly into three biological processes: biotic/abiotic stress responsiveness, phytohormones, and plant growth/development. The majority of *ELP4* genes included the following *cis*-acting elements: ABRE, AAGAA-motif, TCA-element, ARE, TCA, MYB, LTR, I-box, 02-site, MYB-binding site, CAT-Box, TATA-box, G-box, and Sp1 (**Figure 3**). Stress-related *cis*-elements MYB, LTRs, and ARE, which are associated with drought, low temperature, and anaerobic induction, respectively, were stress-related *cis*-elements that were investigated. I-box, Sp1, Myb-binding site, and G-Box were distributed across the promoter regions (responsible for light).

Phytohormone-related *cis*-elements, such as the TCA-element, TCA, and ABRE, were also found, which are associated with abscisic acid and salicylic acid (**Figure 3** and **Supplementary Table 3**). O_2_-site (related in zinc metabolic control), AAGAA-motif (development-related), CAT-box (associated to meristem expression), and TATA-box (transcription start core promoter) are in the growth/development category. Moreover, Sp1 and TATA-box covered the highest portion, while in phytohormonal response, ABRE contributes the maximum portion. These results indicate that *ELP4* genes had versatile functionality in plants and yeast.

### Functional enrichment analysis

**Figure 4:**
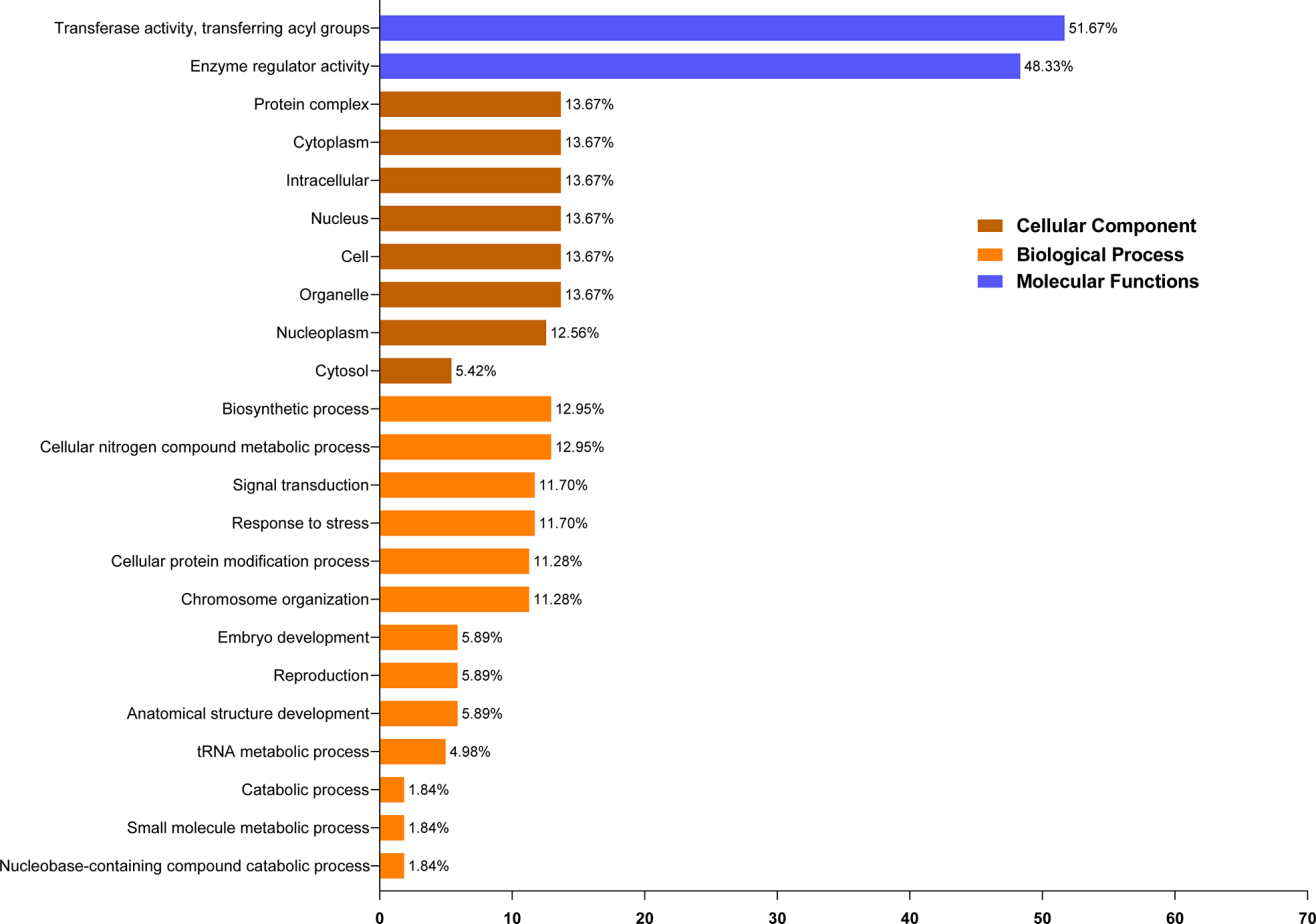
GO enrichment analysis of *ELP4* genes. Based on functions, these are classified into three groups (molecular functions, biological processes, and cellular components) and represented through different colors. GO, gene ontology.

The putative functions of the ELP4 protein are anticipated by utilizing GO annotation analysis. Depending on amino acid similarities, ELP4 proteins were categorized into 23 distinct classes and split into three major ontologies: cellular component, molecular function, and biological process (**Supplementary Table 4**). In molecular function annotation, ELP4 protein was predicted to be most functional in transferase activity (51.67%), followed by enzyme regulator activity (48.33%). In cellular component annotation, ELP4 protein was annotated with protein complex, cytoplasm, organelle, intracellular, nucleus, and cell, with 13.67% followed by nucleoplasm (12.56%) and cytosol (6.68%). Moreover, in biological process annotation, predicted ELP4 protein was annotated with cellular nitrogen compound metabolic process and biosynthetic process along with the same percentage (12.95%), while signal transduction and stress responsiveness contributed equally (11.70%). However, cellular protein modification and chromosomal organization annotated 11.28%, while embryo development, reproduction, and anatomical structure development contributed equally (5.89%). Catabolic, small molecule, and nucleobase-containing compound catabolic processes contribute minimally (1.84%), while the tRNA metabolic process alone contributed 4.98% (**Figure 4**).

### ELP4 functions between *Arabidopsis* and yeast are partially conserved

**Figure 5:**
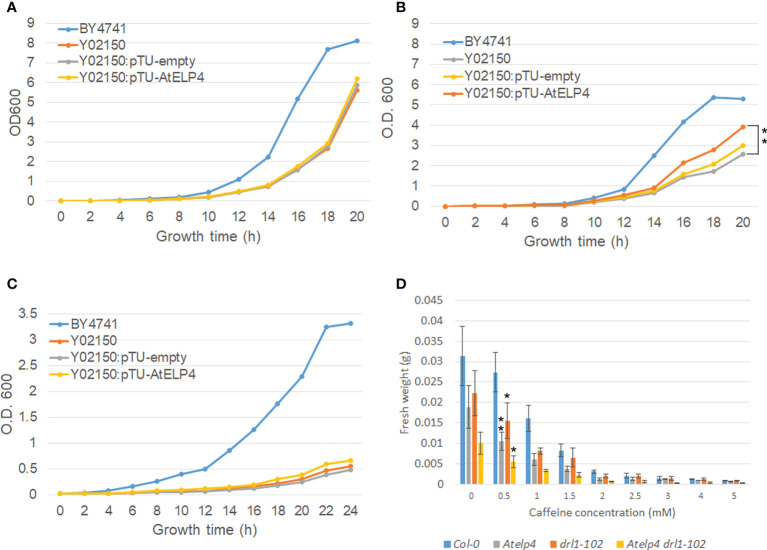
Complementation test of yeast *elp4Δ* mutant and caffeine sensitivity of *Arabidopsis Atelp4* and *drl1-102* single and double mutants. **(A–C)** Complementation of yeast *elp4Δ* mutant, Y02150 by *Arabidopsis ELP4* expression in thermosensitivity and caffeine sensitivity. Wild-type BY4741, *elp4Δ* mutant Y02150, and the empty vector pTU1 or *ELP4*-driven pTU1 introduced Y02150 transgenic lines were grown in YPD media. For test of thermosensitivity, yeast lines were grown at **(A)** 30°C and **(B)** 38°C. Asterisks indicate significant differences compared to Y02150 at 20 h using a Student’s *t*-test (**, p-value <0.01). **(C)** For test of caffeine sensitivity, yeast lines were grown in YPD media containing 7.5 mM of caffeine. Yeast growth was determined by measuring optical densities at 2-h intervals over a period of 20 h using a spectrophotometer at 600 nm. **(D)** Caffeine sensitivity of *Arabidopsis* wild type, and *Atelp4*, *drl1-102*, and *Atelp4 drl1-102* mutants. Plants were grown in MS media containing caffeine concentration in the range of 0 to 5 mM for 21 DAS, and then fresh weight of plants was measured. Ten individuals in each plant were collected, and data are shown as the means with SEs. Asterisks in panels **B** and **D** indicate significant differences compared to no treatment of each plant group using a Student’s *t*-test (*p-value <0.05; **p-value <0.01).

To identify shared functions between yeast and *Arabidopsis*, we performed a complementation investigation utilizing AtELP4 protein in a yeast *elp4Δ* mutant. Yeast *elp4Δ* mutant exhibited resistance toward zymocin and γ-toxin, slow cell growth, thermosensitivity above 38°C, and hypersensitivity toward caffeine (Jablonowski et al., 2001). To confirm the functional complementation of *AtELP4* in yeast *elp4Δ* mutant, we constructed the yeast expression vector *AtELP4* harboring pTU1 and transformed it into yeast BY4741-derived *elp4Δ* mutant, Y02150. Finally, RT-PCR and qRT-PCR were used to validate *AtELP4* expression in yeast *elp4Δ* mutant (**Supplementary Figure 1**). Each transgenic *elp4Δ* mutant containing an empty vector and overexpressing *AtELP4* (Y02150:pTU-empty #1 and Y02150:pTU-AtELP4 #2, respectively) was chosen for further investigation. Compared to the retardation of cell growth of *elp4Δ* mutant Y02150 at normal conditions (30°C), *AtELP4* expression in Y02150 did not rescue to wild type, BY4741 (**Figure 5A**). For a complementation assay for thermosensitivity and caffeine sensitivity, yeast lines grew at 38°C or in caffeine-containing media. Even though it did not fully restore the growth rate of the wild type, Y02150 that expressed *AtELP4* grew faster at 38°C than Y02150 (**Figure 5B**). This result indicates that *AtELP4* expression in Y02150 partially rescued the growth defects at 38°C (**Figure 5B**). However, *AtELP4* expression in Y02150 did not absolutely rescue the sensitivity toward caffeine (**Figure 5C**). These results exhibited that the function of *AtELP4* gene is partially conserved in that of yeast *ELP4* gene.

### Caffeine sensitivity of *Arabidopsis DRL1* and *AtELP4* mutations

As mentioned above, *ELP4* and *KTI12* mutation in yeast triggered the growth defect by caffeine (Jun et al., 2015; Mehlgarten et al., 2017), and caffeine is well known as a cytokinesis inhibitor that disrupts cell plate in dividing cells of plants. To confirm the functional similarity of *DRL1* and *ELP4* between *Arabidopsis* and yeast, we observed the alteration of caffeine sensitivity by single and double *ELP4* and *DRL1* mutants, *Atelp4* and *drl1-102* in *Arabidopsis*. We measured the fresh weight of the plant shoot part that grew on the media containing each caffeine concentration series on 14 DAS. All plants of wild type and single and double mutants showed a decrease in fresh weight depending on caffeine concentration (**Figure 5D**). However, there was a variation in the extent of weight reduction between wild type and mutants. Compared to the normal condition, the fresh weight of each single and *Atelp4 drl1-102* double mutants on the 0.5 mM of caffeine decreased to 70%, 55%, and 55%, whereas that of wild type decreased up to 87% (**Figure 5D**). The hypersensitivity toward caffeine by *DRL1* and *AtELP4* mutation in *Arabidopsis* indicates that *DRL1* and *ELP4* between *Arabidopsis* and yeast were conserved functionally, in the viewpoint of sensitivity of caffeine.

### *Atelp4* mutant had traits similar to *drl1-102* mutant but showed severe phenotype during leaf development

**Figure 6:**
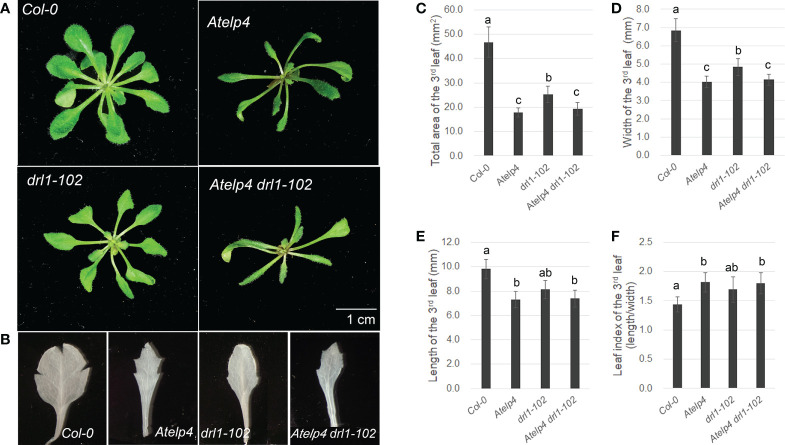
Plant morphology of *Atelp4* and *drl1-102* single and double mutants. Morphology of **(A)** plants on 35 DAS and **(B)** the mature third rosette leaves on 21 DAS of wild type, and *Atelp4*, *drl1-102*, and *Atelp4 drl1-102* mutants. Scale bar indicates 1 cm. **(C)** Total area, **(D)** width, **(E)** length, and **(F)** leaf index (the ratio of length to width of leaf blade) on the mature third rosette leaves in plants on 21 DAS. Leaves were independently collected from 10 plants, and data are shown as the means with SEs. Different lowercase letters in panels **C–F** indicate significant differences between plants, according to ANOVA and Tukey’s multiple range test (p-value <0.01).

We characterized *Atelp4* single mutant and *Atelp4 drl1-102* double mutant of *Arabidopsis* to study the Elongator complex’s role in leaf development. In the previous report, we described that the *drl1-101* mutant formed trumpet-like and filamentous leaves, which had defects in adaxial–abaxial polarity and cell division (Cho et al., 2007). Compared with the wild type (*Col-0*), the *drl1-102* mutant showed narrow and downward-curling (epinastic) leaf blades and an imprecise boundary between the leaf blade and petiole. *Atelp4* mutant also showed narrow leaf blades with severely decreased size and unclear marginal boundary between blade and petiole; *Atelp4 drl1-102* double mutant had a similar phenotype in leaves compared to *Atelp4* single mutant, exhibiting a severe reduction in leaf width and serration in leaf margin (**Figures 6A, B**). The total area of the mature third leaf of *Atelp4* and *drl1-102* reduced up to 54.2% and 39.8% of that of the wild type (**Figure 6C**). Similar results were observed for the *Atelp4 drl1-102* double mutant (39.2% of that of the wild type) (**Figure 6C**). The reduction in leaf width (70.8% to 59.3%) was more severe than the decrease in leaf length (82.8% to 75.1% in the mutants), relative to the wild type (**Figures 6D, E**). Due to more reduced leaf width than leaf length, the leaf index of *Atelp4* and *drl1-102* single and double mutants was higher (1.7, 1.9, and 1.8, respectively) than that of wild type (1.4) (**Figure 6F**), indicating narrower leaf blades in mutants. However, there was no significant difference between the three mutants. Our genetic approaches indicate that *AtELP4* is epistatic to *DRL1*.

### *Atelp4* and *drl1-102* mutants are defective in cell proliferation and the adaxial–abaxial polarity patterning during leaf development

**Figure 7:**
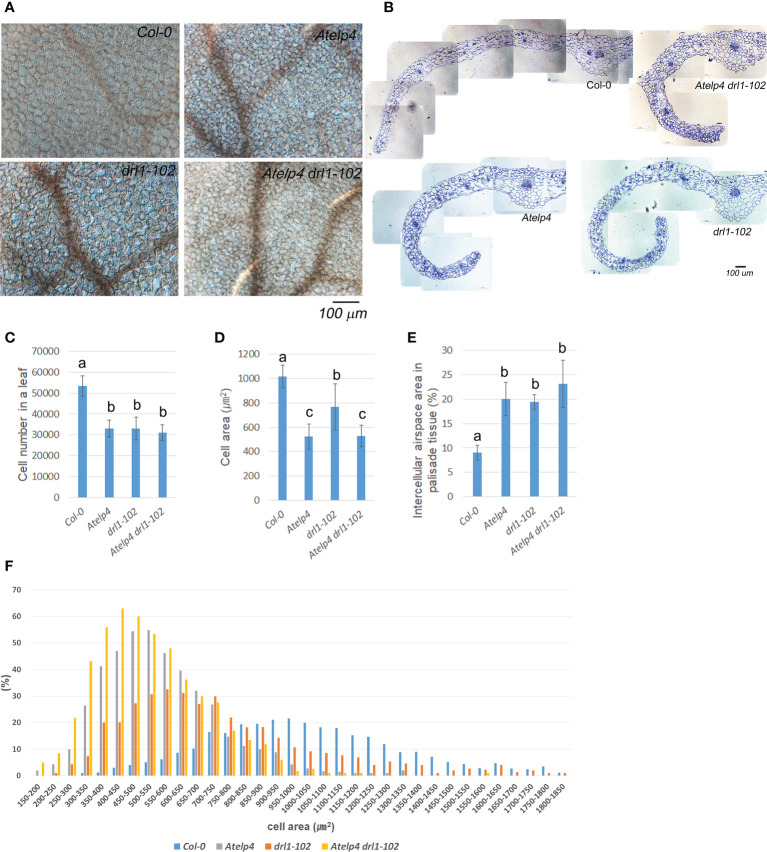
Internal morphology in leaves and statistical analysis of palisade cells from *Atelp4* and *drl1-102* single and double mutants. **(A)** Paradermal images of palisade cells in the mature third rosette leaves in wild type, *Atelp4*, *drl1-102*, and *Atelp4 drl1-102* on 21 DAS. **(B)** Transverse section of the middle region in the mature third leaves. Scale bar = 100 μm. **(C)** Cell number, **(D)** cell size, and **(E)** proportion of intercellular airspace in the mature third rosette leaves on 21 DAS. **(F)** Area distribution of palisade cells in the central region between leaf mid-vein and margin in the widest part of leaf blade. Leaves were independently collected from six plants, and data are shown as the means with SEs. Different lowercase letters in panels **(C–E)** indicate significant differences between plants, according to ANOVA and Tukey’s multiple range test (p-value <0.01).

We used paradermal images and transverse sections to investigate the cellular phenotype at the widest part of the third leaf on the 21 (DAS). This was performed to mitigate the cellular changes in the leaves (Jun et al., 2019). Leaves of *drl1-102* mutant showed reductions in cell number and size, 62.0% and 75.4%, respectively, compared to those of the wild type (**Figures 7C, D**). *Atelp4* mutant showed reduced cell number and size, 61.7% and 51.5% in *Atelp4*, respectively. *Atelp4 drl1-102* mutant also showed a reduction of cell number and size up to 58.2% and 52.0%, respectively, compared to the wild type (**Figures 7A, C, D**). These data indicated that *DRL1* and *AtELP4* positively regulated cell numbers in a similar way. From the viewpoint of cell proliferation and expansion, *AtELP4* was epistatic to *DRL1*.

In the distribution of palisade cell size in leaves, the *drl1-102* mutant showed wider bell-shaped distribution than the wild type (**Figure 7F**). These results indicated that the cell size of *drl1-102* leaves was more irregular than that of the wild type. However, the distribution of cell size in leaves from *Atelp4* and *Atelp4 drl1-102* showed sharper bell-shaped distribution, indicating some suppression processes in cell expansion. The measured cell area of wild type and *Atelp4* and *drl1-102* single and double mutants approximately follows a lognormal distribution with an average of 1,018.0, 767.6, 524.0, and 529.6 μm^2^ with a standard deviation of 92.6, 189.0, 104.3, and 89.0 μm^2^, respectively (**Figure 7F**).

In addition, leaves of each single and double mutant had increased intracellular airspace between palisade mesophyll cells of the adaxial subepidermal layer (**Figures 7B, E**). The proportion of intercellular airspace of *Col-0*, *Atelp4*, *drl1-102*, and *Atelp4 drl1-102* was 11.1%, 17.6%, 18.5%, and 23.4% to the area of the central region between leaf mid-vein and margin in the widest part of the leaf blade, respectively, and showed an increment of intercellular airspace in the adaxial subepidermal layer in each single and double mutants (**Figure 7E**). In detail, intercellular airspace in *drl1-102*, *Atelp4*, and *Atelp4 drl1-102* increased by 215.4%, 221.6%, and 256.1%, respectively, compared to that of the wild type (**Figure 7E**). These findings suggested that in mutants, the palisade’s adaxial side was transformed into an abaxial-like structure.

In the transverse section, mutants exhibited downward curling at the whole leaf blade, an increase in intercellular space/distance on the adaxial side, and a larger portion of phloem tissue in vascular bundles, compared to those of the wild type (**Figure 7B**). Especially, at *Atelp4* and *drl1-102*, each single and double mutant showed remarkable downward curling at the whole part of the leaf blades, whereas the wild type showed downward curling at the marginal part of the leaf blades (**Figure 7B**).

### Decreased chlorophyll content in *Atelp4* and *drl1-102* mutants

**Figure 8:**
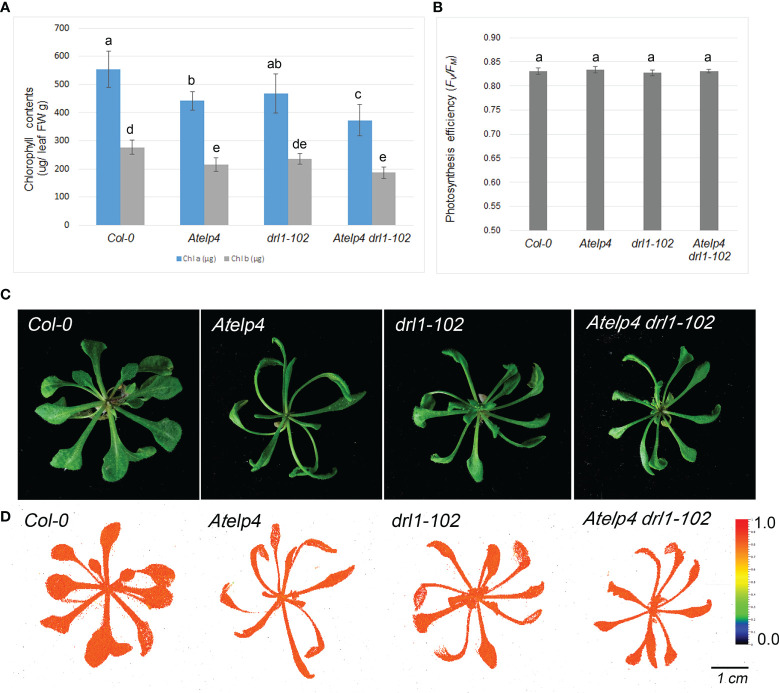
Photosynthesis efficiency and chlorophyll contents of *Atelp4* and *drl1-102* single and double mutants. **(A)** Chlorophyll content. **(B)** Photosynthesis efficiency (*Fv*/*Fm* ratio) (n = 10) measured by FluorCam, **(C)** phenotypes of plants, and **(D)** false color image of photosynthesis efficiency (*Fv*/*Fm* ratio) captured by FluorCam. The color box in panel **D** means photosynthesis efficiency from 0 (minimum value, blue) to 1.0 (maximum value, red). Data are shown as means of eight plants with SEs. Different lowercase letters in panels **A** and **B** indicate significant differences between plants, according to ANOVA and Tukey’s multiple range test (p-value <0.01).

To explore further evidence of the abaxialization of leaves, we measured chlorophyll concentration and photosynthetic efficiency. Total chlorophyll in 1 mg of leaf in *drl1-102*, *Atelp4*, and *Atelp4 drl1-102* mutants was reduced to 53.3%, 48.7%, and 59.5%, respectively, compared to wild type (**Figure 8A**). However, reduction in chlorophyll content had no effect on phenotype and photosynthesis efficiency in mutant leaves, with no variation in *Fv*/*Fm* value when compared to wild type (**Figures 8B, C, D**). Presumably, the residual amount of chlorophyll in mutants was sufficient to support a normal rate of photosynthesis containing light capturing.

The statistical analysis revealed that modifications in *Atelp4* mutant leaves and cells were more severe than those in *drl1-102* single mutant and similar with those in *Atelp4 drl1-102* double mutant. These results indicate that *DRL1* might act upstream of *AtELP4* in two processes: regulation of cell proliferation and adaxial–abaxial polarity establishment.

### *AtELP4* and *DRL1* control the expression of genes that regulate leaf polarity and cell proliferation of leaves

We previously described that *drl1-101* mutant (*No-0* background) showed suppression of cell proliferation and alteration in adaxial–abaxial polarity establishment through RT-PCR.

To verify that *Atelp4* leaves underwent abaxilization in the adaxial side and inhibition of cell proliferation, we next examined the expression level of leaf polarity- or cell proliferation-relative genes by using qRT-PCR.

**Figure 9:**
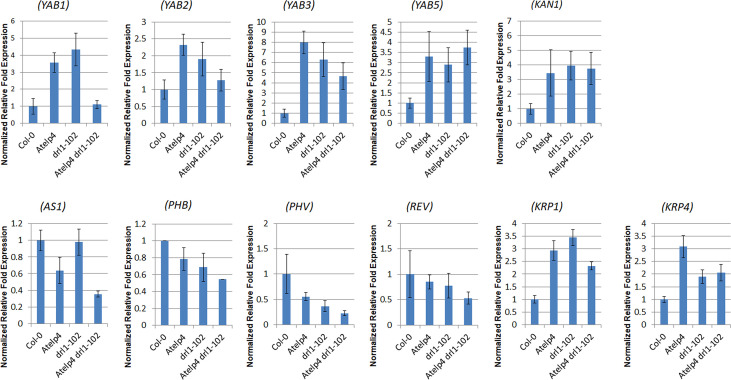
Expression level of leaf polarity- and cell proliferation-relative genes in *Atelp4* and *drl1-102* single and double mutants. Identical amounts of total RNA isolated from 21-day-old rosette leaves of wild type (Col-0), *Atelp4*, *drl1-102*, and *Atelp4 drl1-102* were subjected to qRT-PCR experiments to analyze the expression level of leaf polarity- and cell proliferation-relative genes. The expression level of analyzed genes was normalized by that of *TUB4*. Primer sequences of used genes are described in **Supplementary Table 5**.

*Atelp4*, *drl1-102*, and *Atelp4 drl1-102* mutants showed higher expression levels of the *YAB* family and abaxialization marker, *KAN* genes, and lower expression levels in adaxialization marker genes, *HD-ZIP III* genes, *PHB*, *PHV*, and *REV* (**Figure 9**). In addition, leaf polarity specifying gene, *ASYMMETRIC LEAVES 2* (*AS2*), expression was decreased in *AtELP4* mutation-dependent manner, while leaf abaxial identity specifying genes, *AUXIN RESPONSE FACTOR 4* (*ARF4*), expression was increased in single and double mutants, compared to wild type (**Supplementary Figure 2**).

We also analyzed the expression level of regulating genes of cell proliferation. *Atelp4* and *drl1-102* showed a weak increment in *CYCB1;1* and *CYCD3;1* expression, which is well known as positive cell cycle markers promoting cell division, compared to wild type (**Supplementary Figure 2**). Because these expressions were opposite to expected results in cell proliferation in mutants, we examined the expression level of *KIP-RELATED PROTEINS* (*KRPs*), which act as a cell cycle inhibitor. Single and double mutants displayed increase in *KRP1* and *KRP4* transcripts, compared to the wild type (**Figure 9**). According to the gene expression map of *Arabidopsis* eFP Brower (www.bar.utoronto.ca, Winter et al., 2007), *KRP1* and *KRP4* were strongly expressed in leaves, whereas other *KRP*s expressed much less than *KRP1* and *KRP4* in leaves. Therefore, we speculated that *DRL1* and *AtELP4* might take part in the control of the cell cycle through transcript regulation of cell cycle regulators.

### *AtELP4* and *DRL1* regulate endoreduplication processes during leaf development

During the development of *Arabidopsis* leaves, the cell cycle for cell division and proliferation is known to transit to the endoreduplication cycle repeating DNA replication without entering mitosis (Jun et al., 2013). To understand the function of the Elongator complex on the development of mesophyll cells, we performed a DNA ploidy analysis to examine the nuclear ploidy in the third leaves of *Atelp4* and *drl1-102*.

In 21-day-old wild-type plants (*Col-0*), DNA ploidy distribution in leaves reached an equal portion between the amount of 2C and 4C and the amount of more than 8C. In contrast, *Atelp4* and *drl1-102* showed more portion of the amount of 2C and 4C than that of 8C. 2C and 4C represent the G1 and G2 phases of dividing cells, respectively, while the appearance and accumulation of 8C and more DNA ploidy represent the departure from the cell cycle and activation of endoreduplication (**Supplementary Figure 3**). Hence, this result indicates that endoreduplication is delayed in *Atelp4* and *drl1-102*.

## Discussion

### Conserved functions of ELP4 and DRL1 between *Arabidopsis* and yeast

Elongator in eukaryotes is a highly conserved complex with tRNA modification output that contributes to multiple cellular functions (Nelissen et al., 2005; Nakai et al., 2019). Among the six subunits of the Elongator complex, ELP4 forms a RecA-ATPase-like fold that assembles with ELP5 and ELP6 into a heterohexameric ring (Glatt et al., 2012). Functionally, the yeast (Elp456)_2_ subcomplex specifically recognizes and interacts with tRNA (Glatt et al., 2012). Mutations of yeast Elongator subunits and its associated protein Kti12 caused growth retardation at 38°C and sensitivity to caffeine treatment (Krogan and Greenblatt 2001; Jun et al., 2015; Mehlgarten et al., 2017). Our results showed that *AtELP4* expression partially rescued growth retardation at 38°C of yeast *elp4Δ* mutant (**Figure 5B**). In our previous study, we demonstrated that plant *DRL1* expression also partially rescued growth retardation at 38°C of the yeast *kti12Δ* mutant (Jun et al., 2015), indicating that the gene functions are partially conserved between plant *DRL1* and yeast *KTI12*. Collectively, these cross-complementation analyses suggested that AtELP4 and DRL1 of *Arabidopsis* were closely related in function to their yeast homologs.

In contrast, we found one unknown motif specifically in plant ELP4, although further studies are necessary to prove this motif as a plant-specific binding or catalytic site. Previous research suggested that Kti12 motifs preserved in plant ortholog DRL1 may be involved in cofactor binding (Jun et al., 2015). We revealed that DRL1 is unable to fully replace the function of its yeast counterpart, Kti12, whereas hybrid domain fusion proteins between the two can be based on genetic cross-complementation analysis (Mehlgarten et al., 2017). Non-conserved plant-specific regions in DRL1 have been proposed to facilitate a selective physical or functional interaction with particular Elongator subunits from *Arabidopsis*.

Recently, it was demonstrated that *ELP4* mutation impairs tRNA binding and diminishes Elongator activity, causing neurological defects in human cells (Gaik et al., 2022). Structural studies of mammalian Elongator provided evidence that in higher eukaryotes, Elp123 and Elp456 subcomplexes differ in a cell-type specific function (Gaik et al., 2022). This may be in contrast to unicellular yeast models suggesting species-specific sequence variation in Elongator subunits as a cause of evolutionary differences between lower and higher eukaryotes.

### Elongator complex might control the expression of adaxial–abaxial determinant genes

Our findings show that the distribution pattern of palisade cell size in *drl1-102* mutant leaves is more bell-shaped than in wild type (**Figure 7F**), indicating that the cell size of *drl1-102* leaves was more irregular than that of wild type. In addition, leaves of *Atelp4* and *drl1-102* single and double mutants had increased intracellular airspace between palisade mesophyll cells of the adaxial subepidermal layer (**Figure 7E**). Furthermore, our results showed that loss-of-function mutation in *AtELP4* or *DRL1* induced not only increased expression levels of abaxial determinants, *YAB* and *KAN* families, but also decreased, expressing levels of adaxial determinants, *AS2* and *HD-ZIP III* transcription factors, indicating that Elongator complex might regulate both the repression of the transcription of *YAB* and *KAN* families and the activation of the transcription of *AS1* and *HD-ZIP III* genes. Nelissen et al. (2005) reported that *Atelp3* mutant was abaxialized in the adaxial side of leaves, showing the narrow leaves with less, larger, and more irregularly shaped palisade cells with more intercellular spaces. Kojima et al. (2011) also reported that *AtELP3* represses the expression of abaxial determinant genes, although it was unclear how *AtELP3* regulates the transcription of abaxial determinant genes. These results supported our suggestion of the regulation of the dorsoventrality function by the Elongator complex. Recently, Nakai et al. (2019) reported that the tRNA modification function of Elongator is not required for the control of leaf abaxial–adaxial polarity.

Activation of *YAB1/FIL* transcription is shown to require the interaction of a specific mediator, MONOPTEROS (MP/ARF5), and SWI/SNF complex and recruitment on *YAB* loci at flower primordium initiation (Wu et al., 2015). (Nelissen et al. 2010) suggested based on ChIP analysis that the Holo-Elongator complex did not operate as a general transcription regulator but rather was involved in the acetylation of target genes. Exactly, they reported histone H3 lysine 14 acetylation in the coding regions of auxin repressor *Short Hypocotyl 2* (*SHY2*; called also *IAA3*) and auxin influx carrier *LAX2* genes to be under the control of Holo-Elongator (Nelissen et al., 2010). These findings showed that Elongator-dependent chromatin remodeling may regulate leaf polarity by activating or suppressing the expression of adaxial–abaxial determining genes *via* the auxin signaling cascade. Our results showed a reduction in *AS1* expression in *Atelp4* single and *Atelp4 drl1-102* double mutants, suggesting the Elongator complex affects the expression of *AS1*. Taken together, we suggest that the Elongator complex might act upon a regulatory pathway for the expression of adaxial or abaxial determinant genes. However, the precise mode of Elongator action within the regulation of leaf patterning remains to be shown.

### Elongator may be required for the determination of leaf flatness

Our results proclaimed that *drl1-102*, *Atelp4* single, and *Atelp4 drl1-102* double mutants had defects in the flatness of leaf blades, showing narrow and epinastic leaves (downward curling/curvature of leaf blade margin to abaxial side; downward curling along to the longitudinal axes) (**Figure 7B**). Leaf epinasty/hyponasty (downward curling or upward curling, respectively) was caused by the coordinated anisotropy of growth including cell division and expansion in epidermal, mesophyll parenchyma, and vascular tissues in leaves (Sandalio et al., 2016). The curling of leaves is known to be caused by differences or imbalances of cell proliferation or expansion rate between epidermis or mesophyll cell layers of adaxial and abaxial sides in leaves. In our results, abaxialized in the adaxial side of leaves from *Atelp4*, *drl1-102*, and *Atelp4 drl1-102* mutants caused not only a reduction in cell number and size of palisade mesophyll cells but also an increase in intercellular airspace in the adaxial subepidermal layer, suggesting an alteration in flexibility/strength of the adaxial side (**Figure 7E**).

Consistent with the epinastic phenotype of leaves from *Atelp4* and *drl1-102* mutants, overexpression of *YAB1* or *YAB3* resulted in narrow and epinastic leaves (Siegfried et al., 1999). Loss of function in *AtELP3* also showed narrow and epinastic leaves (Kojima et al., 2011). Additionally, the loss of function of *AS1*, *AS2*, or *HD-ZIP III* and a REV feedback regulator, *ZPR* overexpression, triggered downward curling with abaxialized leaves (Wenkel et al., 2007). These studies supported our suggestion that the Elongator complex may regulate leaf curling.

In contrast, auxin accumulation and gradients are factors controlling leaf epinasty. Exogenous auxin application or genetic alteration of auxin biosynthesis or signaling genes have been reported to present epinastic or hyponastic leaves by the disruption of auxin homeostasis (Keller and Van Volkenburgh, 1997; Keller and Van Volkenburgh, 1998; Kawano et al., 2003). Auxin overproduction caused by a reduction in free IAA or a decrease in auxin responsiveness causes leaf epinasty, according to genetic studies of *rooty* (King et al., 1995), *superroot2* (Delarue et al., 1998), *yucca* (Zhao et al., 2001), and *iaaM* (Romano et al., 1995), whereas elevated auxin level or increased auxin response causes leaf hyponasty from the genetic studies of *icu6* (Pérez-Pérez et al., 2010) and *iamt1-D* (Qin et al., 2005). *iamt1-D*, gain-of-function *indole-3-acetic acid* (*IAA*) *carboxyl methyltransferase1* (*IAMT1*), converts free IAA to methyl-IAA *in vitro*, showing that reduction of free IAA has narrow and hyponastic leaves, whereas RNAi transgenic plants had epinastic leaves (Qin et al., 2005).

Although we do not yet have direct evidence of auxin level changes or response to auxin in leaf morphogenesis in *Atelp4* mutant, our results presented that Elongator might be a linker between auxin response and the establishment of leaf polarity. These complicated multiple functions enable the Elongator to play an important role in auxin signaling (Nelissen et al., 2010, Leitner et al., 2015).

### Elongator function in tRNA modification may be required for proper leaf development

Modification at the anticodon wobble position (U_34_) of tRNA^Lys^, tRNA^Glu^, and tRNA^Gln^ is in charge of fine-tuning mRNA translation efficiency and rate (Nakai et al., 2019). It is reported that the plant Elongator cooperates with two *UBIQUITIN-RELATED MODIFIER 1* (*URM1*)-like proteins involved in sulfur modification of tRNA at U_34_ (Nakai et al., 2019). Lack of URM1-like proteins or AtELP3 exhibited an increase in intercellular airspace, a decrease in chlorophyll contents, and a delay of endoreduplication, suggesting the importance of U_34_ modification in leaf morphogenesis (Nakai et al., 2019). Similarly, our results showed that *Atelp4* and *drl1-102* single and double mutants had increased intercellular airspace and decreased cell number. *Atelp4* and *drl1-102* mutants also showed reduced chlorophyll contents and a delay of endoreduplication in leaves (**Figure 8A** and **Supplementary Figure 3**).

In summary, we suggest that AtELP4 as part of the Elongator complex in *Arabidopsis* plays critical roles in cell proliferation, leaf flatness, and establishment of leaf polarity through a collaboration with DRL1 during leaf morphogenesis. While the tRNA modification function clearly is important for proper leaf morphogenesis, the Elongator complex may also be required to contribute to leaf development in plants. Further studies will have to await the molecular mechanism and mode of action of individual Elongator subunits to elucidate Elongator’s diverse and versatile functions in plants.

## Data availability statement

The datasets presented in this study can be found in online repositories. The names of the repository/repositories and accession number(s) can be found in the article/**Supplementary Material**.

## Author contributions

K-HC, RS and G-TK conceived and designed the study. SEJ, MAM, RS, and G-TK wrote the manuscript. SEJ, MAM, TYH, and YSK performed the investigation and SEJ, MAM, and G-TK analyzed data. All authors reviewed the results and approved the final version of the manuscript.

## Funding

This research was supported by the National Research Foundation of Korea (NRF) grant funded by the Korean government (MSIT) (No. 2021R1A2C1006572), by the Basic Science Research Program through the NRF funded by the Ministry of Education (No. 2020R1A6A1A03047729 and No. 2020R1I1A1A01054294), and by the Green Fusion Technology Program funded by Ministry of Environment.

## Conflict of interest

The authors declare that the research was conducted in the absence of any commercial or financial relationships that could be construed as a potential conflict of interest.

## Publisher’s note

All claims expressed in this article are solely those of the authors and do not necessarily represent those of their affiliated organizations, or those of the publisher, the editors and the reviewers. Any product that may be evaluated in this article, or claim that may be made by its manufacturer, is not guaranteed or endorsed by the publisher.

## Supplementary material

Supplementary Figure 1 Expression level of *AtELP4* and *ScELP4* in yeast wild type (BY4741), *elp4Δ* mutant (Y02150), three lines of pTU1-empty vector harboring *elp4Δ* mutant (#1, #5, and #8), and two lines of pTU1-AtELP4 harboring *elp4Δ* mutant (#2 and #29) by **(A)** semi qRT-PCR and **(B)** qRT-PCR. To confirm *ELP4* silence of Y02150, the expression level of endogenous *Saccharomyces cerevisiae ELP4* (*ScELP4*) was estimated. The expression level was normalized to that of *ScACT1*.

Supplementary Figure 2 Expression level of *AtELP4*, *DRL1*, and other genes by qRT-PCR in *Atelp4* and *drl1-102* single and double mutants. Identical amounts of total RNA isolated from 21-day-old seedlings of wild type (Col-0), *Atelp4, drl1-102*, and *Atelp4 drl1-102* were subjected to qRT-PCR experiments to analyze the expression level of leaf polarity- and cell proliferation-relative genes. The expression level of analyzed genes was normalized by that of *TUB4*. Primer sequences of used genes are described in SUPPLEMENTARY TABLE 5.

Supplementary Figure 3 Distribution of DNA ploidy on the mature 3^rd^ rosette leaves of plants on 21 DAS.
