# Supplementary figures and images for: AtELP4 a subunit of the Elongator complex in Arabidopsis, mediates cell proliferation and dorsoventral polarity during leaf morphogenesis
Source: Front Plant Sci. 2022 Oct 21;13:1033358. doi: 10.3389/fpls.2022.1033358 (PMC9634574; doi:10.3389/fpls.2022.1033358)

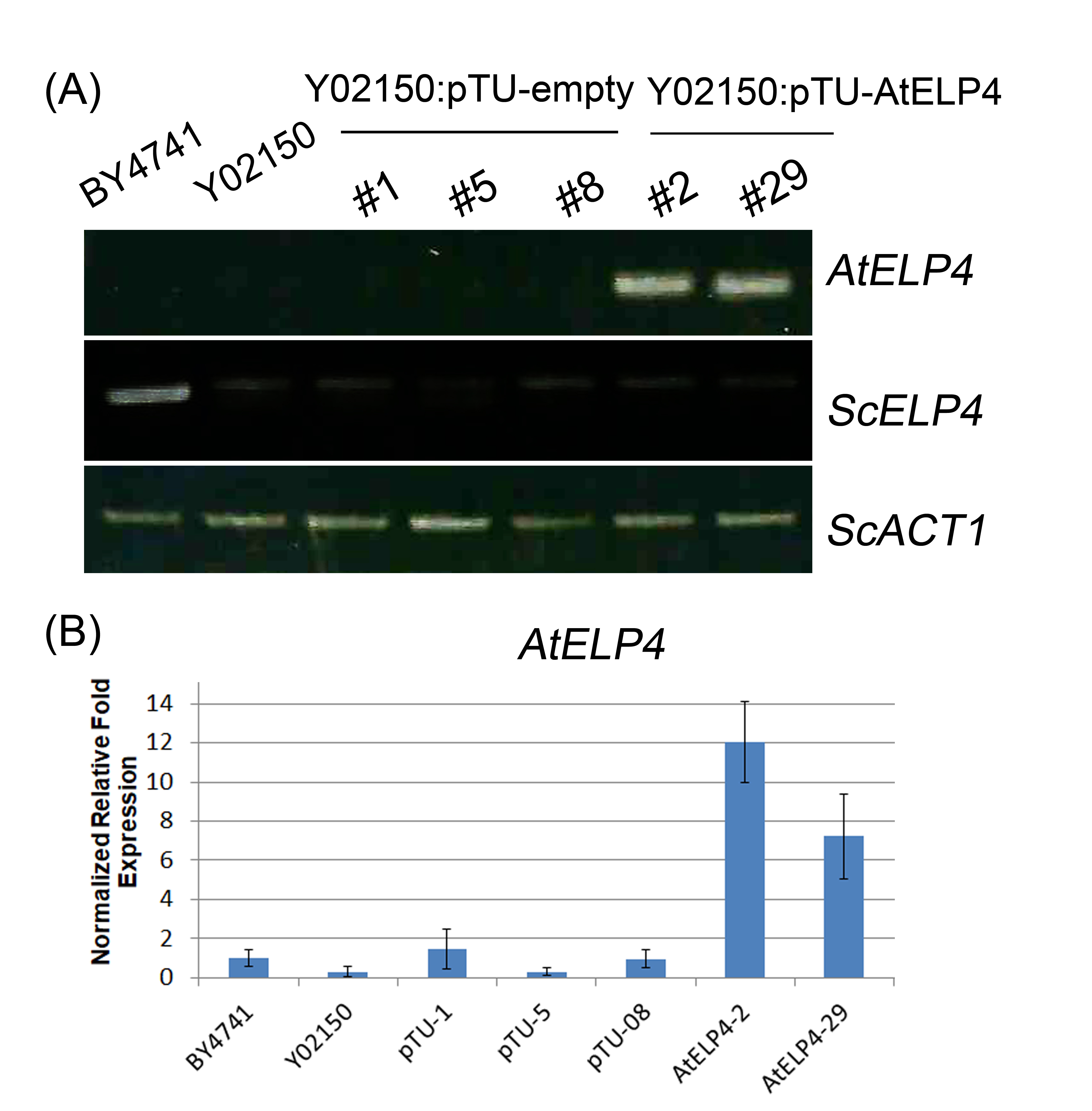

Supplement: Supplementary Figure 1 — Expression level of AtELP4 and ScELP4 in yeast wild type (BY4741), elp4Δ mutant (Y02150), three lines of pTU1-empty vector harboring elp4Δ mutant (#1, #5, and #8), and two lines of pTU1-AtELP4 harboring elp4Δ mutant (#2 and #29) by (A) semi qRT-PCR and (B) qRT-PCR. To confirm ELP4 silence of Y02150, the expression level of endogenous Saccharomyces cerevisiae ELP4 (ScELP4) was estimated. The expression level was normalized to that of ScACT1. [file Image_1.jpeg]

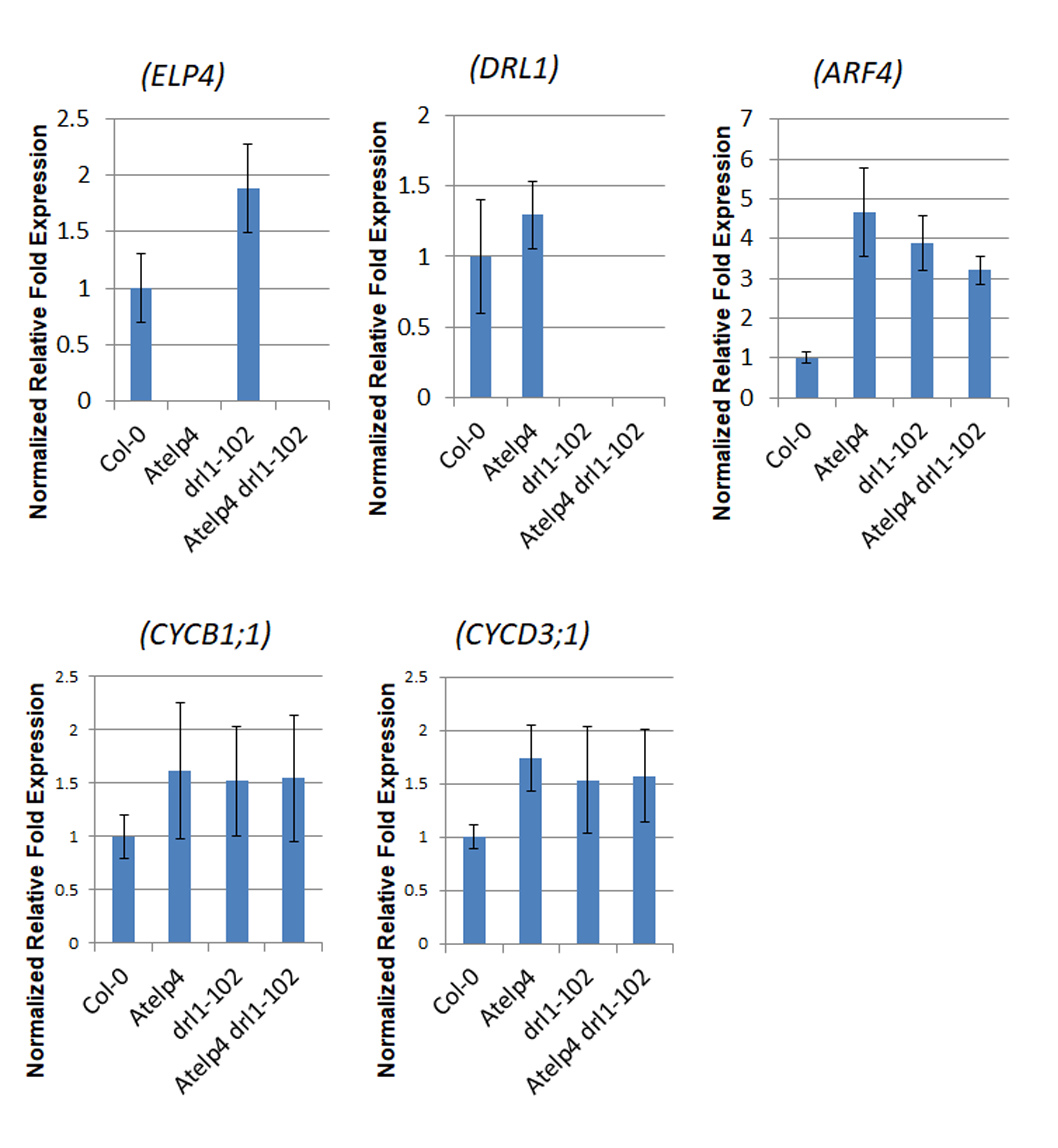

Supplement: Supplementary Figure 2 — Expression level of AtELP4, DRL1, and other genes by qRT-PCR in Atelp4 and drl1-102 single and double mutants. Identical amounts of total RNA isolated from 21-day-old seedlings of wild type (Col-0), Atelp4, drl1-102, and Atelp4 drl1-102 were subjected to qRT-PCR experiments to analyze the expression level of leaf polarity- and cell proliferation-relative genes. The expression level of analyzed genes was normalized by that of TUB4. Primer sequences of used genes are described in SUPPLEMENTARY TABLE 5. [file Image_2.jpeg]

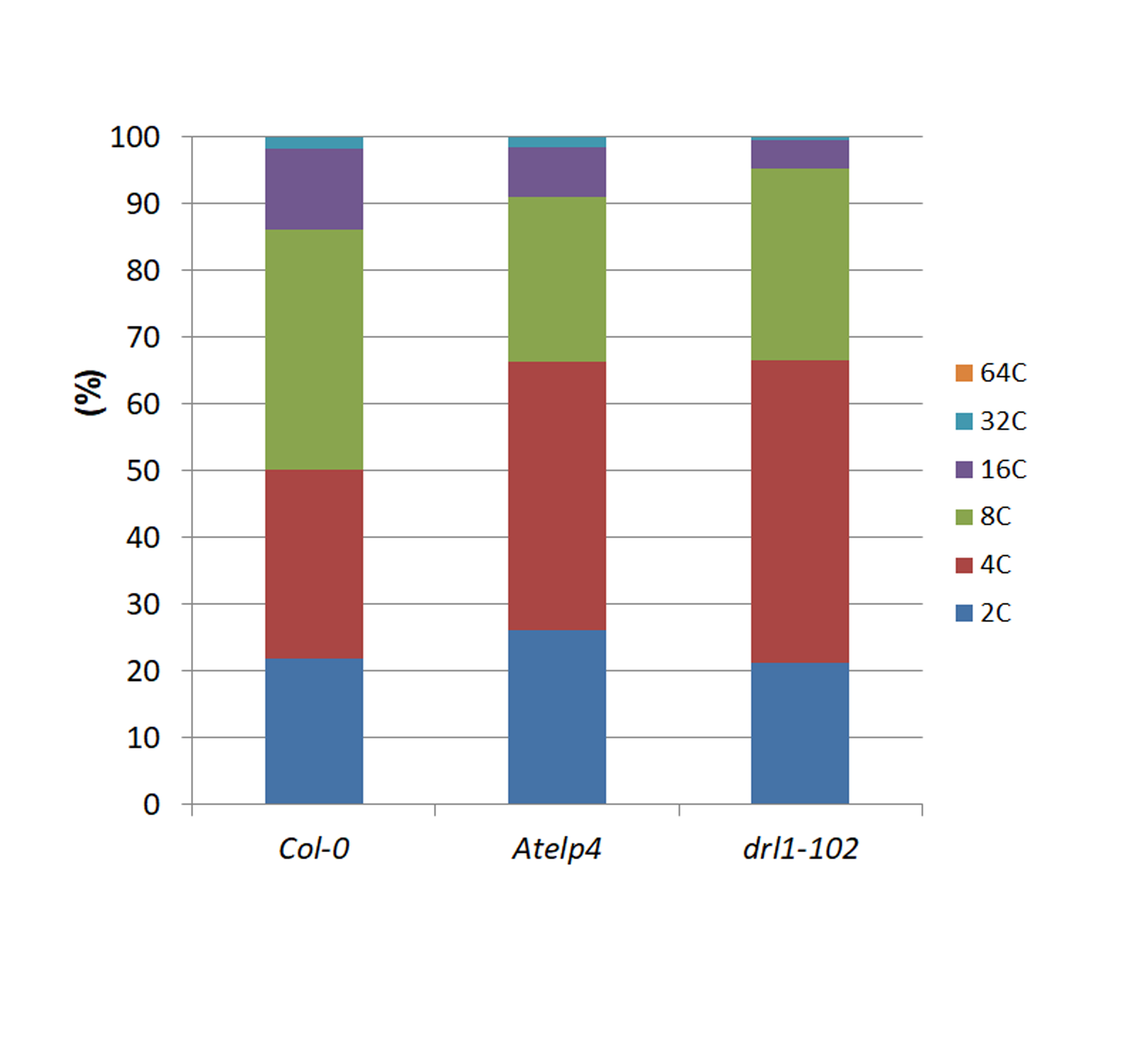

Supplement: Supplementary Figure 3 — Distribution of DNA ploidy on the mature 3rd rosette leaves of plants on 21 DAS. [file Image_3.jpeg]
