# Supplementary material for: AtELP4 a subunit of the Elongator complex in Arabidopsis, mediates cell proliferation and dorsoventral polarity during leaf morphogenesis
Source: Front Plant Sci. 2022 Oct 21;13:1033358. doi: 10.3389/fpls.2022.1033358 (PMC9634574; doi:10.3389/fpls.2022.1033358)
Supplement: Supplementary file 8 [file Table_5.docx]

**SUPPLEMENTARY TABLE 5.** Primer sequences for this study.

| Name | Sequence | Purpose |
| --- | --- | --- |
| DRL1-forward | GCTGAGAAGAATCTGAGAGG | T-DNA insertion |
| DRL1-reverse | CTCCAAACTCTCTGTTCAAG | T-DNA insertion |
| ELP4-forward | CACCGCTGCACCAAACGTTC | T-DNA insertion |
| ELP4-reverse | GCCAGTGAGGAGTTTCTCCA | T-DNA insertion |
| ELP4-forward | CACCATGGCTGCACCAAACGTTCGTAG | cloning |
| ELP4-reverse | TCAAAAATCTAGTGCTCCGG | cloning |
| DRL1-forward | GCTGAGAAGAATCTGAGAGG | qRT-PCR |
| DRL1-reverse | CTCCAAACTCTCTGTTCAAG | qRT-PCR |
| ELP4-forward2 | ATG GAA GAT CCT GAA GCA CC | qRT-PCR |
| ELP4-reverse2 | TGA TGC AGG ATG CGG CAA AGT AC | qRT-PCR |
| KAN1-forward | CCGGCTACAACAACGCTTACC | qRT-PCR |
| KAN1-reverse | CTGCAAATGGCTCTTCACA | qRT-PCR |
| KAN2-forward | AAGGAACTAGATGGAAAGTGCTCAA | qRT-PCR |
| KAN2-reverse | TTAGTGAGATCGACCCAGAG | qRT-PCR |
| YAB1-forward | GCTATGTCCAATGCAACTTT | qRT-PCR |
| YAB1-reverse | TTCTTGGCAGCAGCACTAAA | qRT-PCR |
| YAB2-forward | TCAAACCTCAGCTCCTCCCA | qRT-PCR |
| YAB2-reverse | CTTTTGGCCTGCAACTGACT | qRT-PCR |
| YAB3-forward | ACTTCTCATCTACGGACCAG | qRT-PCR |
| YAB3-reverse | TCAGCCATGAGTCCAAAGTG | qRT-PCR |
| YAB5-forward | ACGCCCTAATTTCCAGGTAAC | qRT-PCR |
| YAB5-reverse | GTTGCTCAGTTATGGTACGAG | qRT-PCR |
| PHB-forward | TGATGGTCCATTCGATGAGC | qRT-PCR |
| PHB-reverse | TCTAAACTCACGAGGCCGCA | qRT-PCR |
| PHV-forward | TCCCAATACGGTAGCTCA | qRT-PCR |
| PHV-reverse | GATGCAGCAGAATAGGCA | qRT-PCR |
| REV-forward | ACCACCGTGAGAGAAGCAGT | qRT-PCR |
| REV-reverse | CAGAGAGCTTCCGGTTTACG | qRT-PCR |
| ARF4-forward | CGCTTAAATCATTCCCGCAAT | qRT-PCR |
| ARF4-reverse | ACTTGTTGGCTTGGTAAGCAAAG | qRT-PCR |
| CYCB1;1-forward | ACCTCGCAGCTGTGGAATATGTG | qRT-PCR |
| CYCB1;1-reverse | CGGGTTTAGCTCGAATCGGACATGC | qRT-PCR |
| CYCD3;1-forward | GCAAGTTGATCCCTTTGACC | qRT-PCR |
| CYCD3;1-reverse | CAGCTTGGACTGTTCAACGA | qRT-PCR |
| KRP1-forward | CGA AAT TGA TGA CGG AGA TG | qRT-PCR |
| KRP1-reverse | GGC TTC TCC TTC TCG AAA TC | qRT-PCR |
| KRP4-forward | AAGACAGTTTGTCACGCAGC | qRT-PCR |
| KRP4-reverse | TGGTTGTTCGTTCACAGGAT | qRT-PCR |
| TUB4-forward | AGAGGTTGACGAGCAGATGA | qRT-PCR |
| TUB4-reverse | CCTCTTCTTCCTCCTCGTAC | qRT-PCR |
| ScACT1-F1 | GTA CCA CCA TGT TCC CAG GTA TT | Semi- or qRT-PCR |
| ScACT1-R1 | ACACCATCACCGGAATCCAA | Semi- or qRT-PCR |
| ScELP4-F1 | CGTGGAGAGATGAGAGTTTT | Semi qRT-PCR |
| ScELP4-R1 | TTAATAGTCTAAAGATATCT | Semi qRT-PCR |
| LBal | TGGTTCACGTAGTGGGCCATCG | T-DNA insertion |
